# Cardiovascular Risk and Metabolic Syndrome Characteristics in Patients with Nonfunctional Pituitary Macroadenoma

**DOI:** 10.1155/2018/2852710

**Published:** 2018-08-27

**Authors:** Guadalupe Vargas-Ortega, Baldomero González-Virla, Lourdes Balcázar-Hernández, Oriana Nieto-Guzmán, Ana Pamela Garrido-Mendoza, Marco Antonio Flores-Maya, Victoria Mendoza-Zubieta

**Affiliations:** Endocrinology Service, Hospital de Especialidades, Centro Médico Nacional Siglo XXI, Instituto Mexicano del Seguro Social, Mexico City, Mexico

## Abstract

**Context:**

An elevated incidence of type 2 diabetes and cardiovascular disease (CVD) has been reported in patients with nonfunctional pituitary macroadenoma (NFPMA). There is no information about metabolic syndrome and cardiovascular risk in patients with NFPMA in our population.

**Objective:**

Analyze the metabolic syndrome (MetS) components and estimate cardiovascular risk in patients with NFPMA.

**Design and Setting:**

Retrospective study, at the tertiary care center.

**Patients and Methods:**

71 patients with NFPMA treated according to a preestablished multimodal protocol.

**Main Outcome Measures:**

Prevalence of diabetes, hypertension, high cholesterol, obesity, and cardiovascular risk and its relationship with the clinical and biochemical characteristics.

**Results:**

The prevalence of diabetes, hypertension, high cholesterol, and obesity at diagnosis was 30%, 27%, 48%, and 85% and did not change upon the last visit. The prevalence of MetS changes from 54 to 48% (*p* = 0.001). NFPMA patients showed a significant increase risk for high total cholesterol (SMR 1.68, 95% CI 1.28–2.17, *p* = 0.001) and diabetes (SMR 3.19, 95% CI 2.19–4.49, *p* = 0.01). According to Globorisk, the male gender was an evidence of high CVD before (81% versus 18%, *p* = 0.01) and after (72% versus 28%, *p* = 0.01) multimodal treatment.

**Conclusion:**

A high prevalence of cardiovascular and metabolic disease and a high cardiovascular risk were evidenced in patients with NFPMA, especially in men. Risk factors such as the personal history of hypertension and dyslipidemia could explain the foregoing, so the control and treatment of metabolic parameters and cardiovascular risk should be an integral part of the follow-up of these patients.

## 1. Introduction

Nonfunctional pituitary macroadenoma (NFPMA) is a benign lesion of the pituitary gland with a diameter larger than 1 cm, which represents approximately 30% of all pituitary tumors. NFPMA do not secrete pituitary hormones that leads to a clinical syndrome; however, they are related to headache, visual field defects with or without decreased visual acuity (mass effects of the tumor), and hypopituitarism. The transsphenoidal surgery is the treatment of choice in these patients; nevertheless, some cases require adjuvant therapy with postoperative radiotherapy [1].

An elevated incidence of type 2 diabetes and CVD, such as acute myocardial infarction and cerebral stroke, has been reported in patients with NFPMA [2–5].

Also, CVD can be related to hypopituitarism and imperfections of hormone replacement strategies, sleep-wake cycle alterations, circadian rhythm dysregulation, and obesity related to hypothalamic dysfunction, all of these being associated with lesion or destruction of the hypothalamic nucleus in large suprasellar lesions [6–9].

A study of 145 patients with NFPMA showed a prevalence of arterial hypertension in 75.9% of patients, fasting hyperglycemia in 31.7%, an elevated abdominal circumference in 53.1%, low high-density lipoprotein cholesterol (HDL-C) in 29%, and hypertriglyceridemia in 46.9%; 41% of patients presented with metabolic syndrome. In this study, patients with NFPMA had an increased risk of low HDL-C (SMR 1.59, 95% IC 1.13–2.11), hypertriglyceridemia (SMR 2.31, 95% CI 1.78–2.9), and metabolic syndrome (SMR 1.6, 95% CI 1.22–2.02), compared to the general population.

A high prevalence of MetS in the general population was reported in our country, which conditions an increased risk of cardiovascular disease; however, the presence of metabolic diseases and cardiovascular risk in patients with pituitary adenoma has not been described. The aim of our study was to analyze the MetS components and estimate the cardiovascular risk in patients with NFPMA upon diagnosis and after multimodal treatment.

## 2. Methods

### 2.1. Patients

In this study, we included 71 patients with evidence of nonfunctional pituitary macroadenoma remnant and multimodal treatment (one or more surgeries and adjuvant fractionated conformal radiotherapy), enrolled in the clinic of pituitary adenomas at Hospital de Especialidades, Centro Médico Nacional Siglo XXI in Mexico City. At the initial and the last visit, we recorded the weight (kg) and height (meters), as well as the waist circumference (cm). A single investigator, using the same calibrated instruments, performed all the anthropometric measurements. Waist circumference (WC) was determined at the middle point between the inferior rim of the last costal arch and the superior rim of the anterosuperior iliac spine. Metabolic parameters (glucose, insulin, glycosylated hemoglobin (HbA1c), triglycerides, total cholesterol (TC), high-density lipoprotein cholesterol (HDL-C), and low-density lipoprotein cholesterol (LDL-C)) were evaluated at diagnosis and upon the last visit (after the multimodal treatment). MetS was defined according to the National Cholesterol Education Program (NCEP) expert panel on detection, evaluation, and treatment of high blood cholesterol in adults (Adult Treatment Panel III), and patients are said to have MetS if they have three or more of the following: WC greater than 102 cm in men and 88 cm in women, blood pressure higher than 130/85 mmHg or antihypertensive drug medication, fasting triglycerides higher than 150 mg/dL, HDL-C less than 40 mg/dL in men and 50 mg/dL in women or a previously treated dyslipidemia, and fasting glucose over 100 mg/dL or diabetes treatment [10]. Patients with diabetes were on treatment with metformin and/or glyburide as oral antidiabetic drugs or insulin (glargine or NPH insulin). Patients with hypertension received enalapril, losartan, or amlodipine. Patients with dyslipidemia received atorvastatin and/or bezafibrate. We used the Globorisk score to calculate cardiovascular risk at diagnosis and upon the last visit after multimodal treatment. Globorisk is a prediction equation of 10-year risk of fatal CVD that can be recalibrated and updated for use in different countries including Mexico; this score can be used to identify individual patients who are at high risk of cardiovascular disease and thus need treatment. Globorisk includes gender, diabetes diagnosis, smoking, systolic blood pressure, and total cholesterol as variables. A score ≥ 10% represents a high fatal CVD risk in low-income and middle-income countries compared with high-income countries [11, 12].

Hypocortisolism was defined as morning cortisol levels below to 3 *μ*g/dL. Central hypothyroidism was defined as free thyroxine (FT4) levels lower than 0.5 ng/dL, regardless of thyroid-stimulating hormone (TSH) concentration. Central hypogonadism was defined as estradiol levels below 20 pg/mL in women and testosterone levels below 300 ng/dL in men. Hyposomatotropism was defined as insulin growth factor (IGF-1) level below the lower limit of normality established for age and gender. Panhypopituitarism was diagnosed if three or more pituitary-hormonal axes were affected. All patients with hypopituitarism received hormone replacement therapy with levothyroxine, prednisone, and estrogen/progestin or testosterone according to the deficit. The replacement dose of daily levothyroxine was calculated at 1.6 mcg/kg body weight per day. Prednisone was indicated at a dose of 5 mg orally once daily (with adjustments of doses during illness, surgery, or another stress situation). Conjugated equine estrogens (0.625 mg/d) plus progestin (medroxyprogesterone acetate 2.5 mg/d) or testosterone enanthate (250 mg/month) were indicated according to gender. Patients with hyposomatotropism did not receive growth hormone replacement on any moment at follow-up [9]. At the last check-up, the adequate hormonal replacement was determinate when free thyroxine (FT4) levels were at a normal range according to the assay method and testosterone levels higher than 300 ng/dL in men. Patients with multiple endocrine neoplasia type 1 were excluded. Our local ethical committee approved the study, and all patients signed an informed consent letter upon diagnosis.

### 2.2. Radiation Protocol

Fractionated conformal radiotherapy was applied by a linear accelerator, 2–2.5 Gy daily for 3 weeks, with a total dose of 52 Gy (range 50–57).

### 2.3. Biochemical Analysis

For biochemical determinations, 6 mL of blood was collected in BD Vacutainer tubes (BD Franklin Lakes, New Jersey, EEUU) and was centrifuged at 3150 ×g for 15 minutes in an Allegra X-22 centrifuge (Beckman Coulter Inc., EEUU) to obtain the serum, which was analyzed with a glucose measuring kit, total cholesterol (TC), HDL-C, triglycerides, and insulin from the COBAS brand (2010 Roche Diagnostics, Indianapolis, EEUU) with a photocolorimetry technique by a spectrophotometer Roche Modular P800 (2010 Roche Diagnostics, Indianapolis, EEUU). For the HDL-C determination, the samples were treated with modified enzymes by polyethylene glycol and dextran sulfate and were analyzed with the same technique. The determination of glycated hemoglobin (HbA1c) was made by immunoanalysis with turbidimetric inhibition, using the COBAS brand kit (2010 Roche Diagnostics, Indianapolis, EEUU). The LDL-C levels were obtained by the Friedewald formula: LDL‐C (mg/dL) = CT (mg/dL) − [(HDL‐C (mg/dL) + triglycerides (mg/dL))/5], as long as the triglyceride concentration was not greater than 400 mg/dL. The anterior pituitary hormones such as testosterone, estradiol, cortisol, and free T4 were quantified with commercially available tests.

### 2.4. Statistical Analysis

The continuous variables are described as mean ± standard deviation (SD) or median and interquartile range (IQR) according to their distribution. For the categorical variables, proportions were used (expected frequency, prevalence). To establish the association between the continuous variables, the Student *t*-test, Mann–Whitney *U* test, or Wilcoxon signed-rank test were used, and for the categorical variables, the *χ*^2^ test was used. We made a multivariate logistic analysis to establish the magnitude of association and calculate SMRs. To establish a statistically significant association, the *p* value < 0.05 was considered. The statistical package SPSS Statistics version 17 and STATA version 11 were used.

## 3. Results

### 3.1. Clinical and Tumor Characteristics

The studied population consisted of 71 patients (mean age 53 ± 13.7 years, 36% women). The median of follow-up from baseline diagnosis of NFPMA to the last visit was 10.2 ± 4.8 years. Fifty-five patients (78%) had a suprasellar tumor with cavernous sinus invasion. Thirty patients (44%) had a single surgery with a transsphenoidal approach, 22 patients (31%) had two surgeries, and 18 patients (25%) had three or more surgeries; most of which were performed through a transcranial approach. All patients had progression of the remnant on follow-up magnetic resonance and received fractionated radiotherapy as an adjuvant treatment. The mean of waiting time between surgery and radiotherapy application was 10 years (interquartile range [IQR] 5–12).

The initial tumor volume at diagnosis of NFPMA was 14130 mm^3^ (IQR 5233–24031). Before radiotherapy, the volume was 1601 mm^3^ (IQR 697–538). Tumor volume decreased to 1124 mm^3^ (IQR 382–2638) in 3 years (*p* = 0.002) and to 816 mm^3^ (IQR 92–1866) in 5 years after radiotherapy (*p* = 0.01). A Δ of 785 mm^3^ in tumor volume reduction at 5 years was observed compared to preradiotherapy volume (reduction of 50%, *p* = 0.01).

The hormonal deficiencies were analyzed at the moment of the diagnosis and in the last check-up, and this analysis found the following proportions: for hypothyroidism, 86.7% versus 92% (*p* = 0.001); for hypocortisolism, 62% versus 78% (*p* = 0.001); for hypogonadism, 67% versus 76% (*p* = 0.001); for hyposomatotropism, 89% versus 94% (*p* = 0.002); and for panhypopituitarism, 67.8% versus 78% (*p* = 0.001) (Table 1).

All patients with a hormone deficit had an appropriate hormonal replacement with levothyroxine, prednisone, testosterone, or estrogen/progestin during all the clinical follow-up, according to the case. The growth hormone was never replaced in patients with hyposomatotropism.

At the last visit, no cases of optic neuritis or central nervous system tumors were evidenced.

#### 3.1.1. Anthropometric and Metabolic Characteristics

The weight and BMI at diagnosis of NFPMA were 76.2 ± 13.4 kg and 29.3 kg/m^2^ (IQR 26.5–31.4), respectively; after multimodal treatment, a weight of 74.7 ± 13.6 kg and a BMI of 29 kg/m^2^ (IQR 26.4–32) were evidenced at the last visit, which was not statistically significant. The 47% of patients had obesity at NFPMA diagnosis versus 40% after multimodal treatment (*p* = 0.001). At diagnosis, the mean of WC was 92.1 ± 12.6 cm in men and 89.6 ± 10.3 in women; at the last visit, WC was 91.9 ± 13.5 in men and 90.3 ± 11.0 in women, which was not statistically significant.

At NFPMA diagnosis, the fasting glucose was 94 mg/dL (IQR 86–99), fasting insulin 13.7 UI/dL (IQR 8.1–25), HOMA-IR 3.0 (IQR 1.8–6.4), HbA1c 6.3% (IQR 5.8–6.5), systolic blood pressure 117 ± 14 mmHg, and diastolic blood pressure 74.6 ± 9.4 mmHg. The total cholesterol was 210 ± 46 mg/dL, HDL-C 43 mg/dL (IQR 35–52), LDL-C 121.8 ± 50 mg/dL, and triglyceride 212 ± 126 mg/dL.

After multimodal treatment, the fasting glucose was 96 mg/dL (IQR 85–115), fasting insulin 16 UI/dL (IQR 7.5–31.5), HOMA-IR 4.9 (IQR 1.9–16.6), HbA1c 6% (IQR 5.7–7.4), systolic blood pressure 114 ± 14.5 mmHg, and diastolic blood pressure 70 ± 15.5 mmHg. The total cholesterol was 199 ± 35 mg/dL, HDL-C 45 mg/dL (IQR 38–60), LDL-C 120 ± 31 mg/dL, and triglyceride 171 ± 67 mg/dL; triglycerides were the only variable with a statistical significance after multimodal treatment (*p* = 0.02) (Table 2).

At the diagnosis of NFPMA, arterial hypertension was found in 27% of patients, CVD in 28%, type 2 diabetes in 30%, and mixed dyslipidemia in 65%. After multimodal treatment, these comorbidities remained in the same proportions. In the group of patients with diabetes, women were more affected than men (58% versus 42%, *p* = 0.003). MetS was evidenced in 52% of patients at diagnosis. The proportion of MetS decreased from 54% to 48% at the last visit (*p* = 0.001). There was no difference in the presence of MetS between patients with hyposomatotropism and patients without hyposomatotropism (41% versus 44%, *p* = 0.68).

The multivariate analysis for diabetes risk showed an OR of 4.2 (95% CI 0.84–1.13, *p* = 0.08) for the woman gender, an OR of 4.9 (95% CI 1.09–22, *p* = 0.03) for personal history of hypertension, an OR of 5.37 (95% CI 1.03–27, *p* = 0.04) for personal history of dyslipidemia, and an OR of 0.74 (95% CI 0.06–8.18, *p* = 0.80) for hyposomatotropism.

Compared to the general population (data from the ENSANUT 2016) [13], NFPMA patients showed a significant increase risk for total cholesterol elevation (SMR 1.68, 95% CI 1.28–2.17) and diabetes (SMR 3.19, 95% CI 2.19–4.49) without a statistical significance for hypertension (SMR 1.05, 95% CI 0.7–1.51) and overweight/obesity (SMR 1.18, 95% CI 0.96–1.43).

Only one patient (with diabetes and hypertension) had a stroke at five years of follow-up (patient received radiotherapy 2 years before CVD).

#### 3.1.2. Cardiovascular Risk

We used the Globorisk score to determine the cardiovascular risk in our population. Patients were stratified in three groups according to the Globorisk score: patients with a risk below 3%, a risk between 3–10%, and a risk above 10%. More than a third of patients had a high cardiovascular risk (≥10 points) at diagnosis and continued with the same score during the follow-up. In gender analysis, there was evidence of high CVD (risk ≥ 10%) in men versus women, before (81% versus 18%; *p* = 0.01) and after multimodal treatment (72% versus 28%, *p* = 0.01) (Table 3).

## 4. Discussion

In this study, we report the characteristics of metabolic syndrome and the cardiovascular risk (according to the Globorisk score) in patients with NFPMA at diagnosis and after multimodal treatment.

Many studies have demonstrated a high prevalence of metabolic syndrome, a decrease in HDL cholesterol levels, and hypertriglyceridemia in patients with NFPMA; hypertension, hyperglycemia, or increased waist-hip ratio had not been reported [2–5].

The increased prevalence of metabolic disorders and CVD could be associated with the extension of large suprasellar tumors to adjacent structures, like the hypothalamus, conditioning a dysregulation in energy balance, disturbed circadian rhythm, sleep irregularities, imbalances in the regulation of body temperature, obesity, heart rate and/or blood pressure imbalance, and feeding alterations due to hypothalamic dysfunction [6–9].

Other related variables could have imperfections of hormone replacement strategies or the absence of hyposomatotropism replacement. The mortality in patients with NFPMA is greater than that in the general population and is related to the increase in cardiovascular, respiratory, and infectious diseases, mainly in women. There is no consensus of variables that predict mortality in NFPMA; however, the advanced age at the moment of the diagnosis and the elevated doses of long-term glucocorticoid replacement therapy could be associated with it. [13].

In our country, the National Health Survey of 2016 reported a prevalence of 9.4% for type 2 diabetes and 25.5% for hypertension in the general population [14].

We observed that the prevalence of diabetes among patients with NFPMA remains higher than that in the general population (30% versus 9.4%), which could increase the presence of CVD. The high prevalence of diabetes could be related to the size of tumor at diagnosis as well as to the delay on diagnosis (approximately 3 years), with the subsequent delay of initial surgery treatment.

A suprasellar extension of tumor with visual field defects was observed in 78% cases; we do not discard the possibility of hypothalamic dysfunction due to suprachiasmatic/paraventricular nucleus damage secondary to tumor invasion or compression.

Hypopituitarism was evidenced in more than 2/3 of patients, which could result in an increased risk and/or worsening of metabolic diseases.

There are no studies in the Mexican population about metabolic syndrome or cardiovascular risk in patients with NFPMA. It would be expected that, in the absence of hormonal hypersecretion, there would be no clinical syndromes or metabolic disorders; however, we demonstrated an increased prevalence of diabetes in patients with NFPMA compared to the general population (30% versus 9.4%, *p* ≤ 0.05), especially in women; this was not evident for hypertension (27% versus 25.5%, *p* = 0.73).

We found a similar prevalence of MetS at the diagnosis of NFPMA with respect to other series (44%); however, after multimodal treatment, a decrease in MetS (52% versus 48%, *p* = 0.02) and a decrease in triglycerides (*p* = 0.02) were evidenced, probably associated with lifestyle changes and nutritional control during the clinical follow-up.

Although Framingham and SCORE are widely used for the cardiovascular risk evaluation [15-17], these scores are not standardized for our population, which limits its application in the Mexican and Latin population. Globorisk is a validated cardiovascular risk score in our country. According to this score, the cardiovascular risk in the Mexican population is higher than that in other countries, with an evidence of high risk (more than 10%) in 15% of men and 11% of women [11, 12]. In our series, a high cardiovascular risk (>10%) was evidenced at diagnosis and after multimodal treatment (45% versus 35%) in both genders. The cardiovascular risk was greater in the NFPMA patients than in the general population, mainly in men.

The limitation in our study is the difficulty to start growth hormone replacement therapy due to the high cost in our health system, which could influence the metabolic outcomes; however, the diabetes multivariate predictor model did not show a statistical significance when hyposomatotropism was controlled. The methodological limitation is the retrospective nature of our study and the lack of a control group, which could condition an increase of bias; a prospective or a case-control study could improve the quality of the inferences.

## 5. Conclusions

The prevalence of diabetes, metabolic syndrome, and high cardiovascular risk (according to the Globorisk score) among patients with NFPMA remains higher than that in the general population, both at diagnosis and after multimodal treatment, especially in the male gender. Risk factors such as the personal history of hypertension and dyslipidemia could explain the foregoing, so the control and treatment of metabolic parameters and cardiovascular risk should be an integral part of the follow-up of these patients.

## Figures and Tables

**Table 1:** Characteristics of NFPMA patients (*n* = 71).

Age (mean ± DE)	59.6 ± 10.2
Female, *n* (%)	26 (36)
VFD at presentation, *n* (%)	55 (78)
One transsphenoidal surgery, *n* (%)	31 (43.6)
Two or more transsphenoidal surgery, *n* (%)	40 (56.3)
Follow up (yr), mean ± SD	10.2 ± 4.8
Three or more pituitary deficiencies, *n* (%)	38 (67.8)
ACTH deficiency, *n* (%)	43 (62)
TSH deficiency, *n* (%)	59 (86.7)
LH/FSH deficiency, *n* (%)	45 (67)
GH deficiency, *n* (%)	48 (88.8)

VFD: visual field defect.

**Table 2:** Clinical and biochemical characteristics of patients with NFPMA.

	At diagnosis	At the last visit	*p*
Age (y), mean ± SD	54.3 ± 13.7	61.9 ± 10.5	0.01
Weight (kg), mean ± SD	76.20 ± 13	74.7 ± 13.6	0.37
BMI (kg/m^2^), median (IR)	29.3 (26.5–31.4)	29 (26.4–32)	0.49
Waist circumference (cm), mean ± SD	Male: 92.1 ± 12.6	Male: 91.9 ± 13.5	0.32
	Female: 89.6 ± 10.3	Female: 90.3 ± 11.0	0.43
Glucose (mg/dL), median (IR)	94 (86–99)	96 (85–115)	0.20
Insulin (UI/dL), median (IR)	13.7 (8.1–25)	16 (7.5–31.5)	0.57
HOMA-IR, median (IR)	3.0 (1.8–6.4)	4.9 (1.9–16.6)	0.41
HbA1c (%), median (IR)	6.3 (5.8–6.5)	6 (5.7–7.4)	0.89
Systolic blood pressure (mmHg), mean ± SD	117 ± 14	114 ± 14.5	0.53
Diastolic blood pressure (mmHg), mean ± SD	74.6 ± 9.4	70 ± 15.5	0.26
Total cholesterol (mg/dL), mean ± SD	210 ± 46	199 ± 35	0.08
HDL-C (mg/dL), median (IR)	43 (35–52)	45 (38–50)	0.21
LDL-C (mg/dL), mean ± SD	121.8 ± 50	120 ± 31	0.68
Triglycerides, mean ± SD	221 ± 121	171 ± 67	0.02

**Table 3:** Risk stratification according to gender.

Globorisk score	Total group (*n* = 71)		Male (*n* = 45)		Female (*n* = 26)	
	At diagnosis	At the last visit	At diagnosis	At the last visit	At diagnosis	At the last visit
<3 points	15%	15%	45%	36%	54%	63%
3–10 points	39%	49%	50%	65%	50%	34%
>10 points	45%	35%	81%^∗^	72%^∗∗^	18%	28%

^∗^ *p* = 0.01: men versus women at diagnosis, ^∗∗^*p* = 0.01: men versus women after multimodal treatment at the last visit.

## Data Availability

All data generated or analyzed during this study are included in this published article (and its supplementary information files). The database generated during the current study is available with the corresponding author on reasonable request.

## Abbreviations

NFPMA: Nonfunctional pituitary macroadenoma
CVD: Cardiovascular disease
LDL-C: Low-density lipoprotein cholesterol
TC: Total cholesterol
HDL-C: High-density lipoprotein cholesterol
LDL-C: Low-density lipoprotein cholesterol
HbA1c: Glycosylated hemoglobin
HOMA-IR: Homeostatic model assessment of insulin resistance
BMI: Body mass index
MetS: Metabolic syndrome
NCEP: National Cholesterol Education Program
SCORE: Systematic Coronary Risk Evaluation
WC: Waist circumference
FT4: Free thyroxine
TSH: Thyroid-stimulating hormone
IGF-1: Insulin growth factor
VFD: Visual field defect
ACTH: Adrenocorticotropic hormone
LH: Luteinizing hormone
FSH: Follicle-stimulating hormone
GH: Growth hormone
SD: Standard deviation
IQR: Interquartile range
OR: Odds ratio
SMR: Standardized mortality ratio.
